# The news we use: A systematic scoping review of fake news stimuli in psychological research

**DOI:** 10.3758/s13428-026-03220-9

**Published:** 2026-11-04

**Authors:** Theodore C. Webster, Ariana Modirrousta-Galian, Faye C. Skelton, Marina C. Wimmer

**Affiliations:** 1 Psychology, Edinburgh Napier University, Centre for Mind, Creativity, and Environment Research, Edinburgh, UK; 2 Department of Experimental Psychology, University College London, London, UK; 3 School of Psychology, University of Kent, Canterbury, UK

**Keywords:** Systematic review, Fake news, Misinformation, Methodology, Stimuli

## Abstract

Research into fake news has surged in popularity, with over 3,000 publications on the topic each year since 2021. Definitions of what constitutes “fake news” vary across the field of psychology, where source, number, content, and presentation of stimuli may all be different yet still described as “fake news.” Thus, conclusions drawn from fake news research may only generalize to the particular definition or presentation of fake news used in a given study. Relatedly, stimuli may not be available or described clearly enough for reproducibility. To assess the scope of these potential issues, we conducted and reported a PRISMA (Preferred Reporting Items for Systematic reviews and Meta-Analyses) systematic scoping review of three databases including the last 11 years of psychology studies that used fake news stimuli. From the 881 included studies, we extracted and described the number, source, standardization, framing, and availability of fake news stimuli. Our findings suggest a large degree of stimuli variability, with high rates of example and full set availability. Most frequently, research stimuli were about politics and were framed as a social media post, and examples were given in-text. We also evaluated ratios of real to fake news stimuli, where an equal number of real and fake articles was the most frequent ratio. We recommend continued transparency, offering suggestions for open science practices following copyright law and considerations of generalizability regarding fake news in research.

As of September 2026, the term “fake news” has appeared in the title or abstract of over 2,000 publications this year, and we can expect this figure to exceed 3,000 as it has every year since 2021 (data from https://www.dimensions.ai/). Despite the avid scholarly interest in fake news, precisely what is meant by the term remains unclear. This may seem surprising given that we have a dictionary definition of fake news—“false stories that appear to be news, spread on the internet or using other media, usually created to influence political views or as a joke” (Cambridge Dictionary, n.d.)—which aligns with how fake news is typically described in the scientific literature (e.g., Lazer et al., 2018; Tandoc et al., 2018). Nevertheless, operationalizations of the term vary widely across studies.

One issue lies in determining what is and is not false, and thus what constitutes false stories. Indeed, what qualifies information as false (Uscinski et al., 2024)? Does there have to be demonstrable evidence of its falsehood, does it have to contradict the scientific consensus, or does it simply have to be subjectively objectionable? In practice, all three criteria have been used to label content as false, despite each of them representing different standards of judgment. Consider the following three statements: (a) the 2024 Southport stabbings were carried out by a Muslim asylum seeker; (b) human-caused climate change is not happening; and (c) COVID-19 originated in a laboratory. Statement (a) is demonstrably false; the attacker was a British citizen born in Cardiff, Wales, to Christian parents (Casciani & Holt, 2025). Statement (b) contradicts the scientific consensus; while the Earth’s climate is a complex system with natural variability and long-term trends that make it difficult to isolate the impact of specific factors, the overwhelming scientific consensus is that human activities are driving global warming (NASA Science, 2024). Statement (c) remains inconclusive; although much of the scientific evidence suggests COVID-19 had a natural, zoonotic origin, circumstantial evidence exists that supports a laboratory origin (Yoo, 2025). Despite each claim’s distinct epistemic status, they have all at some point been labeled as false by the commentariat (Fraser, 2021; Lewis, 2020; Spring, 2024; Uscinski, 2023).

Falsity is central to the definition of fake news. Therefore, the conceptual vagueness surrounding falsity renders the notion of fake news equally ambiguous, and this is reflected in the scientific literature. Tandoc et al. (2018) reviewed 34 papers published between 2003 and 2017 that used the term “fake news” and found six typologies: news satire, news parody, fabrication, manipulation, advertising, and propaganda. Similarly, Molina et al. (2021) reviewed papers that used the terms “fake news,” “misinformation,” “disinformation,” and “alternative facts” to definitionally unify related research concepts. This review was more open-ended, entailing a snowball approach until saturation was reached in terms of variety in theoretical definitions. Molina et al. identified seven types of online content under the umbrella term of “fake news”: false news, polarized content, satire, misreporting, commentary, persuasive information, and citizen journalism. The multifaceted nature of fake news is further complicated by scrutiny surrounding the appropriateness of its various operationalizations. For example, Allcott and Gentzkow (2017) argued that satire should not constitute fake news since it is unlikely to be misconstrued as factual (but see Klein & Wueller, 2017), and some reviews have explicitly excluded studies that use fake news to refer to satire (Broda & Strömbäck, 2024).

As researchers, we can defer the issue of falsity. For example, we can, and often do, define fake news as content that has been labeled as false by professional fact-checking organizations. Under this operationalization, a claim may later turn out to be true yet still function as fake news within a study because it was designated as false at one point in time. While this approach avoids the need for researchers to adjudicate truth, it nevertheless constitutes a distinct standard of falsity—one grounded in past rather than current epistemic status. We consider it important to acknowledge this limitation despite it being unavoidable when the object of research is defined by truth status.

The broad scope of fake news is evident in the diverse forms in which it can appear online and, consequently, in psychological research. Some studies only use the text from fake news headlines (Maertens et al., 2024), while others include accompanying images (Modirrousta-Galian et al., 2023). The amount of detail presented can also differ—both the headline and the byline of fake news articles can be included (Allen et al., 2024), the headline and the full-text article (Pehlivanoglu et al., 2021), or alternatively just the headline (Pennycook et al., 2021; see Fig. 1 for more examples). Moreover, fake news stimuli can vary in terms of its source, with some studies using artificial fake news stimuli created by the researchers (Pereira et al., 2023) or large language models (Maertens et al., 2024), and others using genuine fake news stimuli circulated online (Allen et al., 2024; Luo et al., 2022; Modirrousta-Galian et al., 2023; Pennycook et al., 2021). Lastly, in addition to content, format, and source differences, studies also vary considerably in the total number and type of news stimuli they use. For example, some studies have used as few as two news stimuli (Koetke et al., 2023), while others have used as many as 40 (Modirrousta-Galian et al., 2025). Furthermore, some studies have only used fake news (Pereira et al., 2023), while others have used a mix of real versus fake news (Luo et al., 2022). All these differences may seem small, particularly when compared with typological distinctions (e.g., satire vs. propaganda), but they introduce an additional layer of variability in the operationalization and study of fake news that must be considered.

**Fig. 1:**
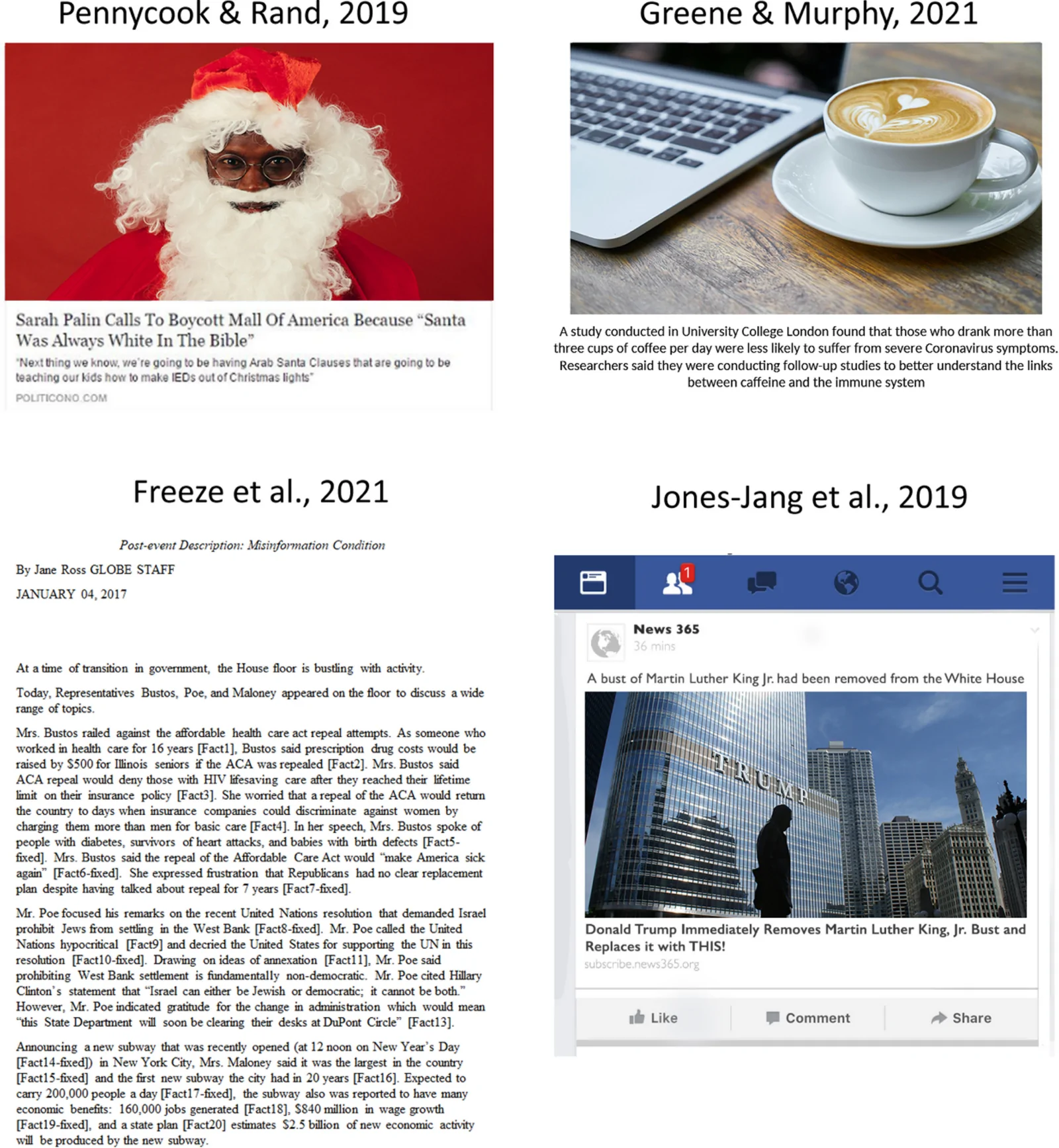
Examples of fake news stimuli in research. *Note*. All text is reproduced from the respective studies, but all images have been replaced with open-access photography due to copyright (Freeze et al., 2021; Greene & Murphy, 2021; Jones-Jang et al., 2019; Pennycook & Rand, 2019)

To explore potential reasons for this variability, we turn to methodological guides on conducting fake news research. Pennycook et al. (2021) recommended that researchers modify or select the news they use to reflect what they are researching. For example, researchers interested in how participants’ political partisanship affects their responses to polarizing news will likely want to pre-test their news stimuli for political alignment, while a study using neutral stimuli may not. Researchers’ perceptions of what can influence their measurement may also change what stimuli are pre-tested for. In the cases of memory and sharing research, news plausibility is more central in memory research (Schincariol et al., 2024) and more peripheral in sharing research (Arin et al., 2023; Sun & Xie, 2024). Therefore, researchers that study false memories for fake news may use pilot studies to select similarly plausible articles and reduce plausibility-related confounds (as in Greene et al., 2021), while researchers interested in fake news sharing may focus on other aspects, such as article topic, personal/social interest, or emotional valence (Shephard et al., 2023; Wenjuan et al., 2023).

Even when researchers take care to control for confounds related to their research area, balancing scores across multiple rating scales while maintaining the intended number of stimuli can be difficult. As a case in point, Delaney et al. (2024) set out to make a set of fake articles about Irish and German people to test in-group/out-group effects of false memories for fake news. These researchers wanted to balance their stimuli on how *common* and *valenced* (i.e., positive or negative) their stereotype-based fake news articles were, due to potential memory effects of these two traits. To that end, they ran a pilot study in which participants rated stereotypes on commonness and valence, with the aim of creating news stories based on these stereotypes that were balanced on these two dimensions. The stereotypes used in their stories, in the end, could not be balanced—when they were equally balanced on the commonness scale, Irish stereotypes were rated as both more positive and more negative than German stereotypes on either side of the valence scale, which the researchers note may have influenced their finding of higher false memories for Irish news overall. Indeed, while Pennycook et al. (2021) recommended using multiple scales to balance articles, they also only matched their news on a single scale (political partisanship) despite collecting ratings for 11 other scales. Including multiple scales is difficult to balance while maintaining stimuli numbers: as an example, we followed this procedure, selecting stimuli with similar ratings on three different scales using both a smaller and larger set of stimuli, and found that closely matching three scales vastly reduced our usable stimuli to ~ 25% of the rated set (this example can be found at https://osf.io/bqac5/?view_only=e8a7d8c177104bec9da7d6899c8b6678).

Furthermore, the presentation of fake news can also vary based on the research question. On the one hand, if you are interested in social aspects of fake news (such as propensity to “Like,” “Share,” or “Comment” on social media, as in MacFarlane et al., 2021, and Yan & Pan, 2023) or source effects (such as reduced sharing of fakes from alternative versus mainstream sources; Di Domenico et al., 2021), you may choose to present your news in an ecologically valid way. Usually, this entails showing the story as if it were a post on social media or an article on a news website (Crum et al., 2024; Jagayat & Choma, 2024). On the other hand, if you are solely interested in the *content* of fake news, you may be wary of the “ecologically valid” presentation style, since participants self-report using context clues (e.g., URL, media company, or author attributions) in news evaluations (Baum & Rahman, 2021; Shen et al., 2019). In this case, you may choose to present news in a source-less, “sterile” way, and forgo the trappings of social media or indications of source altogether (Murphy et al., 2019).

These differences in stimuli, while understandable given the diverse research aims across the field, may present some problems for generalizability. For example, if an effect is found where pre-warnings consistently reduce sharing of political and COVID-19 fake news (as in Pennycook & Rand, 2022), can we assume this same effect will be found in more politically neutral contexts like celebrity fake news? Lawson et al. (2023) presented combined stimuli for COVID-19 and celebrity fake news, finding that people shared the news more often when an online social connection shared the news as well; notably, they interpreted this effect for the full set of stimuli rather than separating COVID-19 from celebrity news. Qualitative research on sharing behavior has found a large variety in reasons for sharing, even when people knew the news was fake (e.g., because it was funny or entertaining; Duffy et al., 2020; Perach et al., 2023); therefore, evaluating sharing behavior by combining COVID-19 and celebrity news could mask item-specific or content-specific sharing rates. In their supplemental material, Lawson et al. (2023) conducted an analysis on the sharing rates between the two types of news and found that COVID-19 news was shared at a significantly higher rate than celebrity news, while sharing of fake celebrity news was related to increased social perception of other sharers. It may be that the danger and health risks of COVID increased overall sharing, while some people also shared celebrity news for social reasons. In such a multivariate and convoluted research area, it may therefore be of interest to delineate findings for certain types or content areas of fake news, rather than generalizing these effects to all fake news.

Differences in stimuli may also affect replicability of fake news intervention research; interventions that work in one context may not work in others. For example, “inoculation” interventions—where participants are exposed to disinformation strategies or messaging styles to create an a priori “immune response” to subsequent viewing of fake news—seem to be robust in certain contexts for reducing sharing of fakes (for health, financial, and political misinformation; McPhedran et al., 2023), but reduced sharing of COVID-19 misinformation in only four of six studies in a systematic review of interventions (Smith et al., 2023). In these cases, the content of the news stories may be a factor in how or why people interact with, share, or believe news. Furthermore, across five studies, Wang et al. (2025) found that when fake news articles were not specifically designed to use the techniques shown during inoculation (as they are in most prominent inoculation research; Roozenbeek et al., 2022a, b), the intervention was not effective.

Fake news research also faces multiple challenges in terms of relevance, replicability, and availability of stimuli. The uniquely short-lived nature of news media means that once-relevant content may quickly be considered outdated (e.g., research conducted with COVID-19 as a misinformation topic has dwindled since 2020; Ciupitu-Plath et al., 2025). At this point, 5 years after the 2020 lockdown, would using the same stimuli as these 2020 studies even be valid replication, considering how distanced from personal relevance and current news these fake articles are? Furthermore, even if researchers wanted to use the same stimuli, they may not be available. In a scoping review of ethical conduct and open science in misinformation research, Greene et al. (2023) found that around only 37% (of 346 total) of misinformation papers across all disciplines detailed their research items (including stimuli). If stimuli are not well described or openly accessible, their content and framing are open to interpretation in replications, which may lead to further differences in the form fake news stimuli take.

Indeed, message stimuli, such as the number of likes on a fake news post, may influence belief in fake news, but diverse presentation framing characteristics makes generalizing findings difficult (Bryanov & Vziatsheva, 2021). In a scoping review of fake news research topics (belief, behavioral intent, etc.), Murphy et al. (2023) reported differences in stimuli, where politics and COVID (post-2020) were the most frequently researched topics, text or text-with-image news stories were the most frequently used formats, and general news or contextless news were the most popular framings in research. Additionally, 45% of studies used both real and fake news, and only 7% of studies reported the ability to discern real news from fake news (Murphy et al., 2023). This review provides information about how fake news stimuli are used in research broadly across all disciplines. However, the framing and format of stimuli may be discipline-specific. For example, computer scientists training an artificial intelligence (AI) model in fake news detection may be scraping the web for hundreds of articles to use as training stimuli, communication researchers may be evaluating responses to specific fake news shared online, and psychology researchers may be using a researcher-fabricated set of normed emotional stimuli.

The presence of many forms of fake news in psychological research raises an important concern about the comparability and generalizability of findings across the scientific literature. When operationalizations and stimuli differ so widely, two studies claiming to investigate fake news may vary significantly. In order to assess the specific breadth of fake news operationalization within one subject area and to keep the review scope manageable, we have focused solely on psychological research. This allows for subject-specific insight into how psychology and behavioral research approaches the topic of fake news, though the multidisciplinary nature of this research topic still includes many different disciplines alongside psychology. Stimuli inconsistency complicates synthesis across studies and may be contributing to emerging issues with replicability in the field, such as failure to reproduce the effects of well-known interventions when tested with different stimuli or in more ecologically valid settings (Modirrousta-Galian et al., 2023; Wang et al., 2025). Addressing the issue of inconsistent operationalizations of fake news in psychological research requires a clear understanding of its scope. To this end, we conducted a systematic scoping review of fake news stimuli used in psychological research over an 11-year period (2014–2025).

## Method

We detail all review processes and exclusions, and provide all material needed to replicate this review. This systematic scoping review follows the PRISMA (Preferred Reporting Items for Systematic reviews and Meta-Analyses) procedure for conducting and reporting a systematic review, with extension for scoping review (Tricco et al., 2018). All search Boolean, list of retained papers, and extraction notes are available on OSF at the following link: https://osf.io/bqac5/?view_only=e8a7d8c177104bec9da7d6899c8b6678. This review was not pre-registered.

Then, as part of a larger systematic scoping review of fake news research methodology using similar methods to the initial pilot scoping review, a second database search was conducted using PRISMA systematic review guidelines (with extension for scoping review).

The search Boolean was designed to find any psychological research paper that may have used fake news as stimuli. Therefore, the Boolean searched for words synonymous with fake news:

$$\begin{array}{l}\mathrm{TS}\;=\;(\mathrm{fake}\;\mathrm{OR}\;\mathrm{false}\;\mathrm{OR}\;\mathrm{misinformation}\;\mathrm{OR}\;\mathrm{disinformation})\\\mathrm{AND}\\\mathrm{TS}\;=\;(\mathrm{news}\;\mathrm{OR}\;\mathrm{media})\\\mathrm{AND}\\(\mathrm{SU}\;=\;\mathrm{Psychology})\end{array}$$

This Boolean was used in a search of three journal databases: Web of Science, PsycInfo, and PsycArticles. Topic search (TS), which includes title, keywords, and abstracts in the search, was used on Web of Science. Abstract search (AB) was used in PsycArticles and PsycInfo, as there was no topic search functionality in these databases. Publication years were set from January 1, 2014, to December 31, 2025. The subject limitation of “SU = Psychology” was removed in the PsycArticles and PsycInfo search, as its inclusion greatly reduced the number of articles found, and this database is already limited to the subject area of psychology. Web of Science (4,092), PsychInfo (2,370), and PsycArticles (113) together returned 6,575 total papers, which were automatically screened using EndNote 20, with 774 automatically removed for not being articles and another 629 automatically removed for being duplicates. In total, 5,172 articles were hand-reviewed, of which a further 170 were duplicates (which were not detected earlier due to slight differences in the automatically retrieved abstracts), 274 were not peer-reviewed articles, and 18 were retracted. Furthermore, 202 retrieved documents did not contain an abstract, and these were manually found through journal searches, where only 19 were found to be journal articles and included in the review.

**Fig. 2:**
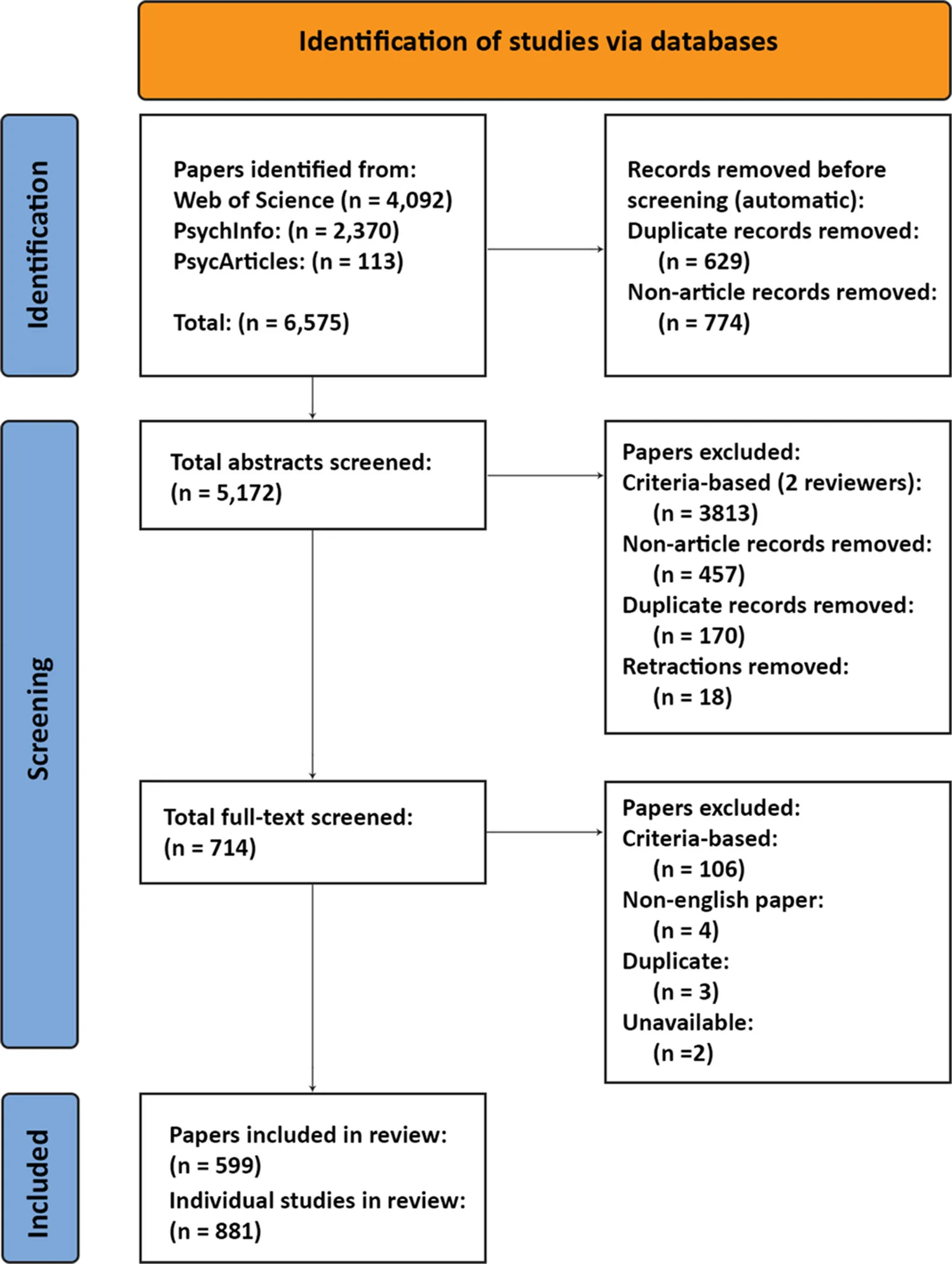
Review process PRISMA flowchart

Two reviewers (TCW and AMG) independently conducted a review of titles and abstracts using the exclusion criteria detailed in Table 1. Papers that were not available in English were also excluded. Any papers for which a reviewer was listed as an author were only reviewed by the other reviewer. In this phase, very high inter-rater reliability (*k* = 0.77) was achieved, with 545 retained papers and 270 disagreements. In cases of disagreement, after reviewer discussion, a further 169 articles were retained, and a total of 714 articles proceeded to full-text review (Fig. 2).

**Table 1:** Exclusion criteria for the expanded review

Exclusion criteria
Did not use fake news articles as stimuli
Did not measure human outcome related to fake news
Did not include any analysis related to fake news (or content analysis only)

The criterion of “human outcome” measures was included to filter for experimental or quasi-experimental research using human participants.

### Data extraction

The full text of each article was downloaded and read, with multi-study papers separated into individual studies. A hand-search was conducted by a single reviewer (TCW) to extract the stimuli source (e.g., fact-checking websites or researcher-created), stimuli number (true and fake news), piloted pre-ratings (e.g., controlling for emotionality or plausibility), and stimuli framing (e.g., presented as a Facebook article or a plain text headline), and example or full set availability. These data were extracted into a Microsoft Excel document, which is available at https://osf.io/bqac5/?view_only=e8a7d8c177104bec9da7d6899c8b6678.

## Results

A total of 714 psychological research articles used fake news as stimuli, of which 171 detailed more than one study that met the inclusion criteria (see Table 2 for the number of papers containing 1–9 studies). A total of 881 studies were included. All percentages reported in this section (other than in Table 2) are out of the number of studies.

**Table 2:** Number of papers containing 1–9 studies meeting inclusion criteria

Number of studies	Number of papers	
	*n*	%
1	425	71.0
2	113	18.9
3	30	5.0
4	15	2.5
5	10	1.7
6	2	0.3
7	1	0.1
9	1	0.1

Pilot studies were not included in this table or extraction. No papers reported 8 studies.

### Stimuli source

Researchers can either find real instances of fake news or they can generate their own. To evaluate how frequently either of these practices have been used in psychology research, any reported stimuli source or generation technique was noted in the extraction (Table 3). Studies that did not clearly report the source of their stimuli (i.e., where the stimuli were selected from or how they were created) were left blank (334 studies, 37.9%). Supplemental materials provided by extracted papers were also searched for source description. The highest frequency of studies (210, 23.8%) used stimuli found on fact-checking sites, of which 119 (13.5%) specified Snopes.com, 37 (4.2%) specified PolitiFact and 24 (2.7%) specified Factcheck.org. News sites were used as a stimuli source in 127 (14.4%). A total of 125 studies (14.2%) used fake news stimuli that the researchers created themselves. Some studies used stimuli from prior research (112, 12.7%), with 35 studies (4.0%) specifically citing a publication including Pennycook as first author as the source of their fake news (Pennycook & Rand, 2019, 2020, 2021; Pennycook et al., 2021).

**Table 3:** Reported sources of fake news

Fake news source	*n*	%
Fact-check sites	210	23.8
News sites	127	14.4
Researcher-created	125	14.2
Prior research	112	12.7
AI-generated	15	1.7
Health providers	14	1.6
Government	12	1.4
Twitter	12	1.4
Facebook	9	1.0
YouTube	5	0.6
Google	4	0.5
Instagram	2	0.2
Weibo	1	0.1
Other	8	0.9
Not specified	106	37.2

Percentages total over 100%, as some papers may have obtained fake news from multiple sources (e.g., used four Snopes.com articles and created the rest).

### Number of stimuli used

To assess the number of stimuli used in psychological fake news research, the reported amounts of real and fake news presented in each study were extracted. Note that the number of stimuli presented may be different from the total number of stimuli, as some studies presented subsets or a portion of a larger set of stimuli. A total of 862 studies (97.8%) clearly reported the number of stimuli shown to study participants. Of these, 542 (61.5%) used both real and fake news in their study, whereas 320 (36.3%) used only fake news (some of these studies showed real *or* fake news depending on experimental conditions, where only conditions showing fake news were extracted to keep comparisons consistent [97 studies, 11%]). Some studies used variable numbers of real and/or fake news across multiple experimental conditions, with 55 studies (6.2%) varying the number of both real and fake news, 23 studies (2.6%) varying only real news, and 3 studies (0.3%) varying only fake news. On average, 8.28 fake articles (*SD* = 9.48, *Mdn* = 6) and 6.50 real articles (*SD* = 10.72, *Mdn* = 3) were used. When looking at ratios of real versus fake news in studies that used both, 315 studies (35.8%) used an equal ratio (1:1) of real and fake news. Conversely, 71 studies (8.1%) used more real news, while 77 (8.7%) used more fake news.

### Stimuli framing

**Fig. 3:**
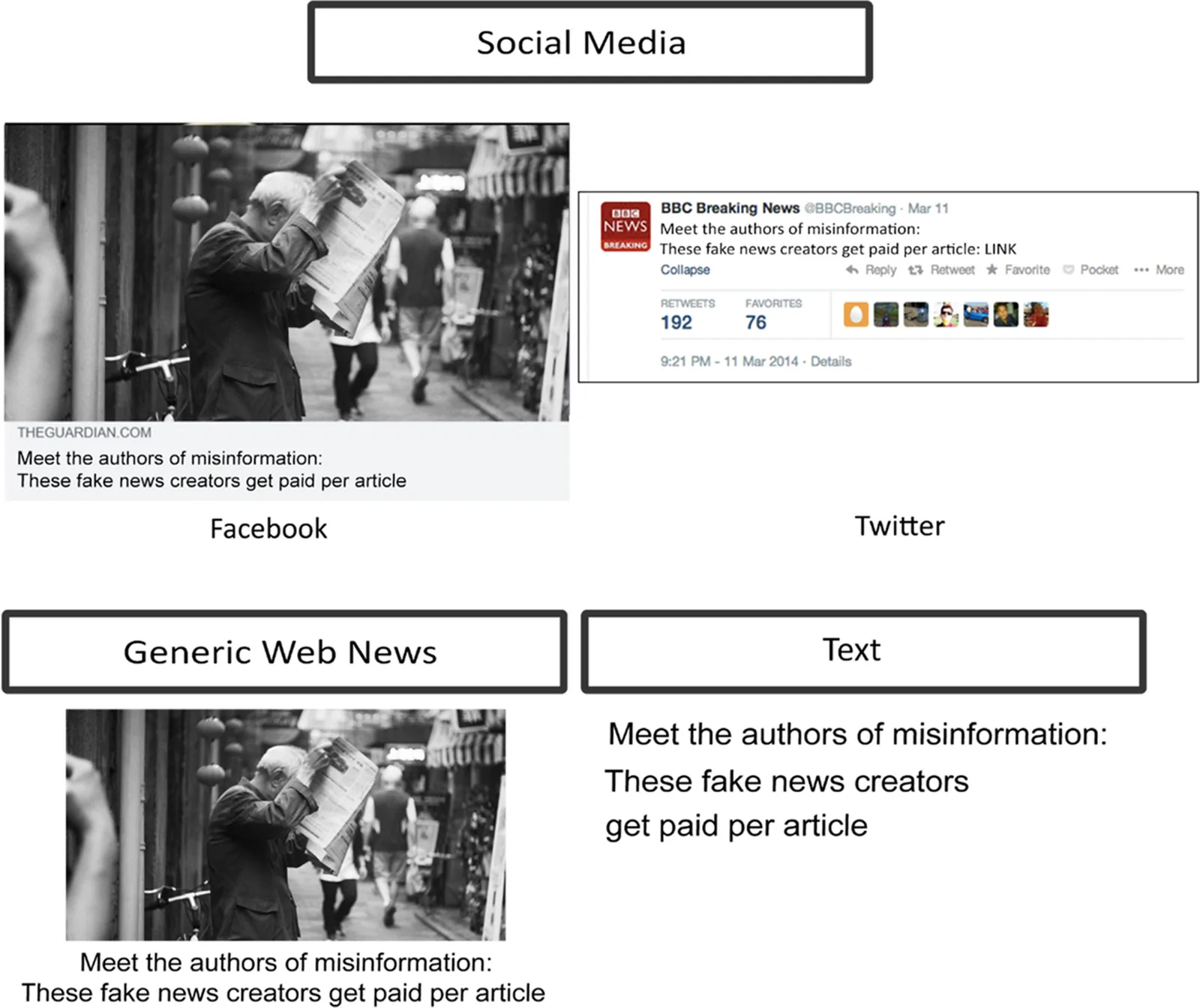
Examples of the top three most prevalent news framing styles. *Note*. Each framing style may be accompanied by “Like,” “Share,” or “Comment” buttons depending on the study. News image adapted from photograph by AG Z via Pexels, available under the open-access Pexels license (2025)

Fake news in research can take many forms, ranging from an “ecologically valid” screenshot of a social media page to a “sterile” text-only news headline. To assess how frequently different presentation styles are used, descriptors of the stimuli framing were extracted from the text and noted on the extraction sheet, with common reported presentation styles categorized by the extractor. Seemingly following recommendations from Pennycook et al. (2021), many stimuli were presented as if they were Facebook newsfeed articles (in 205 studies, 23.3%). A further 146 studies (16.6%) mimicked other specific social media sites like Twitter, Instagram, or TikTok. Generic web news (169 studies, 19.2%) used a similar presentation of a news image and headline without any branded presentation. These can be shown alongside aspects such as likes, favorites, or “stars” to present as brandless social media. In this category, stimuli may also have actually been from Facebook or Twitter, with authors avoiding naming the platforms in text. In some studies, framing was used to present news in an ecologically valid way, while in others it was part of a manipulated condition, such as social engagement by way of likes and shares. Examples of what these three main formats look like are shown in Fig. 3. The fourth most frequently used format, full articles (78 studies, 8.9%), included a full multi-paragraph article or summary of a longer article. Among studies presenting only text, 286 studies (32.5%) presented plain text, and 30 studies (3.4%) presented full-text articles (or briefer full-article summaries). Other framing styles are noted in Table 4.

**Table 4:** Stimuli framing styles used in studies

Stimuli framing	*n*	%
Social media	351	39.8
Facebook	205	23.3
Twitter	100	11.4
Instagram	22	2.5
Weibo	9	1.0
WhatsApp	9	1.0
TikTok	5	0.6
YouTube	1	0.1
Blog	2	0.2
Text	286	32.5
Generic web news	169	19.2
Full article	78	8.9
Full article (text only)	30	3.4
Video	25	2.8
Deepfake video	12	1.4
Audio	6	0.7
Vignette	6	0.7
Web page	5	0.6
Deepfake audio	2	0.2
Other	3	0.3
Unclear	28	3.2

Articles that refer to their presentation as solely “news,” “fake news,” “articles,” or “headlines” without specifying the presentation are listed as “unclear,” as these descriptions were used interchangeably to describe both text-only and text-and-image presentation styles. Percentages total over 100%, as some studies use multiple types of stimuli framing.

### Stimuli topics

To determine the most frequently used topics in psychological fake news research, the topic areas of fake news within the studies were extracted. Topics were coded by one reviewer based on the topics explicitly stated in-text, or were extracted from the full stimuli set if no topic was specified in-text. Extracted text indicating the topic of news stimuli are detailed in the OSF extraction spreadsheet. The topics of the fake news articles used are detailed in Table 5. In 563 studies (63.9%), the fake news stories were intentionally constrained to one news area (a single topic), such as political events, COVID-19, or health. Overall, 146 studies (16.6%) categorized their news into two or three topics, while 162 studies (18.4%) had many different topics (more than four, or one topic per stimuli). Some studies did not specify the topic of their news (18 studies, 2.0%). The most frequently researched topic was political news (273 studies, 31.0%).

**Table 5:** Fake news stimuli topics

Stimuli topic	*n*	%
Political	273	31.0
COVID	194	22.0
Multiple	162	18.4
Health	108	12.3
Neutral	75	8.5
Vaccine	29	3.3
Climate	23	2.6
Business	21	2.4
Crime	17	1.9
Food	15	1.7
Science	15	1.7
Immigration	13	1.5
Conspiracy	7	0.8
GMO [genetically modified organism)]	6	0.7
Religion	3	0.3
Unclear	18	2.0
Other	28	3.2

“Neutral” is a fairly catch-all term in the literature. Neutral news articles may vary by topic but were explicitly described as being politically neutral by the respective studies’ authors. Some neutral news articles were about natural disasters, animals, and celebrities. Studies with multiple topics/no specified topic are listed together due to similar methodologies: when article topics were not constrained, there would often be four or more topics (potentially including political news), occasionally with each individual news article having its own topic. Percentages total over 100%, as some studies specified several topics.

### Stimuli rating and standardization

To assess the quality and consistency of news stimuli, some studies opted to pilot a set of articles to limit potential confounds (e.g., in case varied levels of humor or emotion in news may impact believability or memory). Details of stimuli creation and any pilot studies were extracted from the text (Table 6). A total of 615 studies (69.8%) did not mention or detail any pilot studies for stimuli creation in-text or in their supplemental materials. On the other hand, 266 studies (30.2%) included rated news articles on a variety of measures. Of these rated stimuli, the most common ratings included political alignment (64 studies, 7.3%), familiarity (35 studies, 4.0%), and emotion (28 studies, 3.2%). Studies using an expert selection process (30 studies, 3.4%) may have used rated stimuli but did not specify. It is important to note that rated stimuli do not always mean balanced stimuli. Some studies rated stimuli but did not use ratings in stimuli selection; occasionally, unbalanced stimuli were used in research (i.e., fake news having a lower plausibility score than real news).

**Table 6:** Studies using rated stimuli

Rating used	*n*	%
Total using rated stimuli	266	30.2
Political side	64	7.3
Familiarity	35	4.0
Expert selection	30	3.4
Emotion	28	3.2
Plausibility	27	3.1
Valence	26	3.0
Accuracy	26	3.0
Credibility	24	2.7
Prior research	24	2.7
Belief	23	2.6
Truth	21	2.4
Interest	11	1.2
Content	11	1.2
Informativeness	9	1.0
Value	9	1.0
Validity	9	1.0
Sharing	8	0.9
Surprising	8	0.9
Source	6	0.7
Salience	6	0.7
Readability	6	0.7
Intent	5	0.6
Trust	5	0.6
Humor	5	0.6
Sensationalism	5	0.6
Realism	5	0.6
Arousal	4	0.5
Convincing	4	0.5
Qualitative	3	0.3
Impact	3	0.3
Veracity	3	0.3

Studies that used rated or normed stimuli often used multiple scales.

### Stimuli availability

To assess the transparency and replicability of fake news stimuli in research, any note or explanation in-text of example fake news stimuli or where to find them was extracted. In this case, the example had to align with the stimuli framing, so an example of a generic web news story had to have shown an example of image and headline (see previous section for specific content details). Most studies provided examples either in the text (550 studies, 62.4%) or in an appendix or supplemental material (244 studies, 27.7%), with a total of 794 studies (90.1%) showing an accessible example. Only 57 studies (6.5%) did not provide examples, or examples were specifically mentioned as request-only by the authors. Lastly, 29 studies (3.3%) only showed partial examples (see the Note for Table 7 for an explanation of partial examples).

**Table 7:** Availability of fake news stimuli examples

Example given	*n*	%
Example present	794	90.1
No example	57	6.5
Partial example	29	3.3

Partial examples of stimuli may not have shown a full example of what they described their stimuli to look like (i.e., researchers reportedly used a headline and image but only provided a headline).

To ascertain the availability of full sets of stimuli in psychological research regarding fake news, the full text and any linked or attached supplemental material or appendices were manually searched (Table 8). Most studies provided a full set of stimuli (657 studies, 74.6%). Some studies only provided a part of the verbatim stimuli (73 studies, 8.3%). That is, these studies provided the full list of stimuli but missed some part of the verbatim presentation (e.g., studies that reported using images and headlines may have only reported headlines). A total of 192 studies (21.8%) did not provide their full set of stimuli, wherein 21 (2.4%) mentioned appended or supplementary material that was either not present or available, 20 (2.3%) cited another work as the source but provided no stimuli themselves and did not clarify which ones they used, and 1 (0.1%) was available explicitly by request only.

**Table 8:** Availability of full stimuli set

Full set availability	*n*	%
Full set available	657	74.6
No full set	192	21.8
Missing verbatim	73	8.3

“Missing verbatim” sets were full sets that were not the verbatim stimuli described in the experiment (e.g., a study reportedly showing a Facebook feed with images and headlines but only making the headline text available).

## Discussion

This review demonstrates the variety of stimuli that fall under the umbrella term of “fake news.” Across 881 psychological studies using fake news stimuli, we show that this term can cover many different content areas and presentation formats and can be pre-rated on various dimensions. Furthermore, methodological factors like stimuli sources and numbers can also vary widely. First and foremost, we consider this stimuli variability to be a strength of the literature, as the alternative, that is, using the same stimuli across studies, would severely limit generalizability to real-world settings. However, these differences—and their potential influence on interpretations—may muddle the validity of claims in fake news research. It is therefore important to improve the precision and transparency of stimuli reporting in this research area.

One potential improvement relates to the availability of stimuli. In our review, 86.2% of studies provided at least one example of their stimuli. While this is certainly a step in the right direction, full stimuli availability would constitute better open science practice. A systematic scoping review of misinformation research by Greene et al. (2023) found that only around 50% (out of 346) of papers included supplemental material at all, and only around 35% detailed research items (like stimuli). While this indicates room for improvement regarding the availability of full stimuli sets in misinformation research, our findings showed that a higher percentage of psychology papers shared full stimuli (74.6%).

However, researchers may be wary (or legally incapable) of sharing their news stimuli due to copyright infringement. Our findings indicate that many researchers source their stimuli from fact-checking sites (like Snopes.com) or from prior published research. Therefore, any image included as part of the stimuli may fall under copyright protection. Even when researchers create their own text for fake news stimuli, they may have sourced accompanying images from actual news sources or otherwise copyrighted material. This could cause issues when sharing stimuli, as most journals require permission and copyright attribution for images, even if they fall under Creative Commons or Fair Use, both of which legally allow for use of images in research. For example, the American Psychological Association (APA, 2020) Publication Handbook states that copyright attributions must always accompany images published from other sources, and a letter granting permission for each image must be included when submitting a manuscript. Most major academic publishers have similar policies. Indeed, Springer Nature (2025) lists images taken from the Internet as third-party content and advises authors to obtain written permission from rights holders as early as possible during manuscript preparation. Similarly, Elsevier (2025) states that, unless explicitly available under an open-access license without commercial reuse restrictions or explicitly in the public domain, figures from the Internet require permission. Researchers may therefore need to start requesting copyright permission early in the publication process, and even then, permission is not guaranteed. Nevertheless, there are ways to both retain replicability and follow copyright law. News text—without images—can still be shared if their accompanying images are constrained by copyright. Similarly, especially if image content is a focus of the research, researchers could replace copyrighted images with open-access images to provide more accurate examples, though this should be clearly explained in-text. However, as previously stated, depending on the journal, this may still result in a time-consuming process of copyright permissions.

The problem of reproduceable, generalizable, and copyright-free stimuli is not new to psychological research. In research areas interested primarily in imagery and emotion, creating stimuli sets ad hoc based on the experiment often led to a lengthy piloting process beforehand and reproducibility issues afterwards, mainly due to standardization concerns (Lang et al., 1997). Though several image sets were created to make more reproducible and standardized stimuli (such as the International Affective Picture System; Bradley & Lang, 2017; Libkuman et al., 2007), there was still a lack of open-access material that could be published without per-image copyright attribution and permission. To this end, the Open Affective Standardized Image Set (OASIS) was created, with a main goal of creating a set of images standardized on valence and arousal while being completely available to include in publications under a Creative Commons attribution license (Kurdi et al., 2017). This solves several issues: researchers no longer need to take the time to create and test standardized stimuli and can share used stimuli freely (potentially only needing a single copyright attribution and permission letter). A similar collection of open-access fake news stimuli could be greatly beneficial, though the time-relevant nature of news may make this a unique challenge to address. Potentially, a resource that scrapes the web for recent false news content along with its exposure metadata (e.g., views or retweets) while using ratings (e.g., valence and arousal) to select stimuli could provide a solution for providing a standardized, up-to-date, and representative fake news stimuli pool to select from.

Regardless of copyright complications, the issue remains that the term “fake news” encompasses a wide range of operationalizations. This variability spans multiple dimensions, including content area (e.g., political vs. health-related fake news), presentation format (e.g., social-media-style vs. headline-only fake news), selection criteria (e.g., fake news chosen for plausibility vs. emotionality), and source (e.g., researcher-created vs. real fake news). Each of these dimensions could influence how people respond to fake news, so we consider it essential for researchers to (1) specify or share their news stimuli and (2) consider the implications of these stimuli. Researchers (including us) are already making efforts to do this. For example, Pfänder and Altay (2025) consistently used the term “fact-checked false news” throughout their systematic review and meta-analysis of news judgments. They also identified the limitation that all but three studies in their meta-analysis relied on fact-checked false news; these three studies used false news scraped from the web and checked against ground truths by hired fact-checkers rather than news from fact-checking websites. Participants’ subsequent truth judgments (compared with the ground truth) yielded different results, highlighting the importance of being clear about news selection and the generalizability of findings. Modirrousta-Galian et al. (2026) tested the effects of an inductive learning intervention on news veracity discernment. In a Constraints on Generality paragraph (Simons et al., 2017), the authors restated that they used fact-checked false news headlines and thus only expected their results to be reproduced with the same type of stimuli. Although few journals require a Constraints on Generality section (e.g., *Journal of Experimental Psychology: General*/*Applied* and *Neuropsychology*), we encourage fake news researchers to include such a paragraph regardless of journal guidelines to avoid overgeneralizations.

The importance of avoiding overgeneralizations is difficult to overstate. Consider, for example, inoculation interventions that teach people to recognize manipulation techniques thought to be common in misinformation (e.g., emotional language). Inoculation interventions have been described as psychological vaccines against misinformation and fake news in the academic literature (e.g., Biddlestone et al., 2025; Traberg et al., 2022) and popular press (e.g., van der Linden & Roozenbeek, 2021; Walton, 2017). However, as noted by Wang et al. (2025), the vast majority of research examining the effectiveness of inoculation interventions used synthetic, researcher-created news stimuli reflecting the manipulation techniques targeted by the intervention. When different stimuli were used, namely real-world news stimuli or researcher-created news stimuli containing other manipulation techniques not targeted by the intervention, inoculation treatments produced null effects (Modirrousta-Galian et al., 2023; Wang et al., 2025). By the time this research was published, however, inoculation interventions had already been implemented by technology companies (University of Cambridge et al., 2022,1). This case illustrates an interesting contrast to the broader literature, as inoculation research has typically relied on stimuli that were *insufficiently* diverse, and future studies on intervention effectiveness should focus on using diverse and real-world representative news stimuli. Nonetheless, this example underscores the importance of precise and transparent stimuli reporting without overgeneralization.

In addition to differences in operationalizations, our systematic scoping review revealed considerable variability in the number of stimuli used in psychological fake news research. We found that 61.5% of studies showed participants both real and fake news, while 36.3% only showed fake news. The design choice to use two types of stimuli versus only one type affects the measures and conclusions that can be drawn from a study. For instance, using both real and fake news allows for detection or discriminability measures to be calculated, representing the ability to distinguish between the two types of stimuli, whereas using only fake news precludes such analyses. Without real news stimuli, any improvement in fake news detection could instead reflect a general increase in skepticism. For example, if an intervention lowers veracity ratings to fake news but does not measure veracity ratings to real news, it is impossible to determine whether its effects are specific to fake news or general to all news. Failing to rule out general skepticism effects is an important limitation considering that real news is more prevalent than fake news on social media (Guay et al., 2023).

Relatedly, 35.8% of studies used a 1:1 ratio of real and fake news, 8.1% used a ratio that had more real news than fake news, and 8.7% used a ratio that had more fake news than real news. The ratio of real and fake news can influence participant responses. Altay et al. (2025) found that higher proportions of fake (versus real) news headlines (17%, 33%, 50%, 66%, and 83%) decreased media trust, and while they did not affect participants’ actual ability to distinguish between real and fake news, they made participants more overconfident in their ability to do so. Orchinik et al. (2025) exposed participants either to 80% real news headlines and 20% fake news headlines or vice versa and found that participants were worse at detecting the less prevalent type of news. That is, participants exposed to a higher prevalence of real news were worse at detecting fake news, and participants exposed to a higher prevalence of fake news were worse at detecting real news. A focus on number and ratio of real and fake news is also important for generalizability with the real media landscape, where Allen et al. (2020) found that fake news consumption may be lower than 1% of total consumed news media. Given the impact of design choices pertaining to the number of news stimuli used, it is important for researchers to justify their decisions and discuss their potential implications.

### Conclusion

In this systematic scoping review, we found that the term “fake news” in psychological research is used to describe a wide variety of stimuli that differ in source, topic, and framing. We also found substantial variability in both the number of fake news stimuli used and the procedures employed to select them. While most studies provided full stimuli sets (74.6%), examples of stimuli (90.1%), or framing specifications (96.8%), there is still room for improvement in replication practices regarding stimuli. However, lack of cohesion between open science practices and copyright law may be acting as a barrier to transparency. To circumvent this barrier, we recommend that researchers secure copyright permissions early in the publication process, use open-access stimuli, or provide text-only descriptions of stimuli. Another possible solution is to create an open-access standardized news database like those used in affective imagery research. We also recommend that researchers consider how their choice of stimuli might shape the interpretations and generalizability of their results. Ultimately, the inconsistent operationalization of fake news in psychological research underscores the need for greater transparency around stimuli selection, which is crucial for the reliability and reproducibility of findings in the field.

## Open practices statement

All materials and extraction notes are available at https://osf.io/bqac5/?view_only=e8a7d8c177104bec9da7d6899c8b6678. This review was not preregistered.

## Data Availability

All search Boolean and inclusion/exclusion details required to replicate this review are provided. Extraction notes and reference list for all extracted papers are available on OSF at the following link: https://osf.io/bqac5/?view_only=e8a7d8c177104bec9da7d6899c8b6678

## Footnotes

1 Roozenbeek et al. (2025) did exactly this, conducting two X (formerly Twitter) ad campaigns targeting almost a million users with either a video-based inoculation intervention or a control video. The authors hypothesized that users who saw the inoculation video would engage less with negative emotional content and share less content from unreliable sources than users who saw the control video, but the results showed null effects.

## References

[CR1] Allcott, H., & Gentzkow, M. (2017). Social media and fake news in the 2016 election. *Journal of Economic Perspectives,**31*(2), 211–236. 10.1257/jep.31.2.211

[CR2] Allen, J., Watts, D. J., & Rand, D. G. (2024). Quantifying the impact of misinformation and vaccine-skeptical content on Facebook. *Science*. 10.1126/science.adk3451 38815040

[CR3] Allen, J., Howland, B., Mobius, M., Rothschild, D., & Watts, D. J. (2020). Evaluating the fake news problem at the scale of the information ecosystem. *Science Advances*. 10.1126/sciadv.aay3539 PMC7124954 32284969

[CR4] Altay, S., Lyons, B. A., & Modirrousta-Galian, A. (2025). Exposure to higher rates of false news erodes media trust and fuels overconfidence. *Mass Communication and Society,**28*(2), 301–325. 10.1080/15205436.2024.2382776

[CR5] American Psychological Association. (2020). *Publication manual of the American Psychological Association: The official guide to APA style* (Seventh edition). American Psychological Association.

[CR6] Arin, K. P., Mazrekaj, D., & Thum, M. (2023). Ability of detecting and willingness to share fake news. *Scientific Reports,**13*, 7298. 10.1038/s41598-023-34402-6 PMC10160725 37147456

[CR7] Baum, J., & Abdel Rahman, R. (2021). Emotional news affects social judgments independent of perceived media credibility. *Social Cognitive and Affective Neuroscience,**16*(3), 280–291. 10.1093/scan/nsaa164 PMC7943368 33274748

[CR8] Biddlestone, M., Roozenbeek, J., Suiter, J., Culloty, E., & van der Linden, S. (2025). Tune in to the prebunking network! Development and validation of six inoculation videos that prebunk manipulation tactics and logical fallacies in misinformation. *Political Psychology*. 10.1111/pops.70015

[CR9] Bradley, M., Lang, P. (2017). International Affective Picture System. In: Zeigler-Hill, V., Shackelford, T. (eds) *Encyclopedia of Personality and Individual Differences*. Springer, Cham. 10.1007/978-3-319-28099-8_42-1

[CR10] Brand, A., Allen, L., Altman, M., Hlava, M., & Scott, J. (2015). Beyond authorship: Attribution, contribution, collaboration, and credit. *Learned Publishing,**28*(2), 151–155. 10.1087/20150211

[CR11] Broda, E., & Strömbäck, J. (2024). Misinformation, disinformation, and fake news: Lessons from an interdisciplinary, systematic literature review. *Annals of the International Communication Association,**48*(2), 139–166. 10.1080/23808985.2024.2323736

[CR12] Bryanov, K., & Vziatysheva, V. (2021). Determinants of individuals’ belief in fake news: A scoping review determinants of belief in fake news. *PLoS ONE,**16*(6), e0253717. 10.1371/journal.pone.0253717 PMC8224890 34166478

[CR13] Cambridge Dictionary. (n.d.). Fake news. In *Cambridge Dictionary*. Retrieved May 14, 2025, from https://dictionary.cambridge.org/dictionary/english/fake-news

[CR14] Casciani, D., & Holt, D. (2025, February 25). *Police chief ‘told not to reveal Rudakubana’s faith’*. BBC News. https://www.bbc.co.uk/news/articles/c8d491mqqq5o

[CR15] Ciupitu-Plath, C., Carrell, B. C., & Papadopoulou, E. (2025). University students’ health literacy during the COVID-19 pandemic: A review of observational studies. *Cogent Education*. 10.1080/2331186X.2024.2445349

[CR16] Crum, J., Spencer, C., Doherty, E., Richardson, E., Sherman, S., Hays, A. W., Saxena, N., Niemeyer, R. E., Anderson, A. P., Čeko, M., & Hirshfield, L. (2024). Misinformation research needs ecological validity. *Nature Human Behaviour,**8*(12), 2268–2271. 10.1038/s41562-024-02015-2 39375544

[CR17] Delaney, T., Castillo, L., Friehs, M. A., Buttlar, B., & Greene, C. M. (2024). Us versus them: The role of national identity in the formation of false memories for fake news. *Journal of Experimental Psychology: Applied,**30*(1), 33–47. 10.1037/xap0000498 37902694

[CR18] Di Domenico, G., Sit, J., Ishizaka, A., & Nunan, D. (2021). Fake news, social media and marketing: A systematic review. *Journal of Business Research, 124*, 329–341. 10.1016/j.jbusres.2020.11.037

[CR19] Duffy, A., Tandoc, E., & Ling, R. (2020). Too good to be true, too good not to share: The social utility of fake news. *Information, Communication & Society,**23*(13), 1965–1979. 10.1080/1369118X.2019.1623904

[CR20] Elsevier. (2025). *Permissions*. https://www.elsevier.com/en-gb/about/policies-and-standards/copyright/permissions

[CR21] Fraser, T. (2021, July 16). *Climate crisis is real and stems from human activity*. AP News. https://apnews.com/article/fact-checking-967359390899

[CR22] Freeze, M., Baumgartner, M., Bruno, P., Gunderson, J. R., Olin, J., Ross, M. Q., & Szafran, J. (2021). Fake claims of fake news: Political misinformation, warnings, and the tainted truth effect. *Political Behavior,**43*(4), 1433–1465. 10.1007/s11109-020-09597-3

[CR23] Greene, C. M., de Saint Laurent, C., Murphy, G., Prike, T., Hegarty, K., & Ecker, U. K. H. (2023). Best practices for ethical conduct of misinformation research: A scoping review and critical commentary. *European Psychologist,**28*(3), 139–150. 10.1027/1016-9040/a000491

[CR24] Greene, C. M., & Murphy, G. (2021). Quantifying the effects of fake news on behavior: Evidence from a study of COVID-19 misinformation. *Journal of Experimental Psychology: Applied,**27*(4), 773–784. 10.1037/xap0000371 34110860

[CR25] Greene, C. M., Nash, R. A., & Murphy, G. (2021). Misremembering Brexit: Partisan bias and individual predictors of false memories for fake news stories among Brexit voters. *Memory,**29*(5), 587–604. 10.1080/09658211.2021.1923754 33971789

[CR26] Guay, B., Berinsky, A. J., Pennycook, G., & Rand, D. (2023). How to think about whether misinformation interventions work. *Nature Human Behaviour,**7*(8), 1231–1233. 10.1038/s41562-023-01667-w 37563304

[CR27] Jagayat, A., & Choma, B. L. (2024). A primer on open-source, experimental social media simulation software: Opportunities for misinformation research and beyond. *Current Opinion in Psychology,**55*, 101726. 10.1016/j.copsyc.2023.101726 38048652

[CR28] Jones-Jang, S. M., Mortensen, T., & Liu, J. (2019). Does media literacy help identification of fake news? Information literacy helps, but other literacies don’t. *American Behavioral Scientist,**65*(2), 371–388. 10.1177/0002764219869406

[CR29] Klein, D., & Wueller, J. (2017). Fake news: A legal perspective. *Journal of Internet Law*, *20*(10), 5–13. https://ssrn.com/abstract=2958790

[CR30] Koetke, J., Schumann, K., Porter, T., & Smilo-Morgan, I. (2023). Fallibility salience increases intellectual humility: Implications for people’s willingness to investigate political misinformation. *Personality and Social Psychology Bulletin,**49*(5), 806–820. 10.1177/01461672221080979 35272524

[CR31] Kurdi, B., Lozano, S., & Banaji, M. R. (2017). Introducing the Open Affective Standardized Image Set (OASIS). *Behavior Research Methods,**49*(2), 457–470. 10.3758/s13428-016-0715-3 26907748

[CR32] Lang, P. J., Bradley, M. M., & Cuthbert, B. N. (1997). International affective picture system (IAPS): Technical manual and affective ratings. *NIMH Center for the Study of Emotion and Attention,**1*(39–58), 3.

[CR33] Lawson, M. A., Anand, S., & Kakkar, H. (2023). Tribalism and tribulations: The social costs of not sharing fake news. *Journal of Experimental Psychology. General,**152*(3), 611–631. 10.1037/xge0001374 36892902

[CR34] Lazer, D. M. J., Baum, M. A., Benkler, Y., Berinsky, A. J., Greenhill, K. M., Menczer, F., Metzger, M. J., Nyhan, B., Pennycook, G., Rothschild, D., Schudson, M., Sloman, S. A., Sunstein, C. R., Thorson, E. A., Watts, D. J., & Zittrain, J. L. (2018). The science of fake news. *Science,**359*(6380), 1094–1096. 10.1126/science.aao2998 29590025

[CR35] Lewis, T. (2020, October 12). Eight persistent COVID-19 myths and why people believe them. *Scientific American*. https://www.scientificamerican.com/article/eight-persistent-covid-19-myths-and-why-people-believe-them/

[CR36] Libkuman, T. M., Otani, H., Kern, R., Viger, S. G., & Novak, N. (2007). Multidimensional normative ratings for the International Affective Picture System. *BEhavior Research Methods,**39*(2), 326–334. 10.3758/BF03193164 17695361

[CR37] Luo, M., Hancock, J. T., & Markowitz, D. M. (2022). Credibility perceptions and detection accuracy of fake news headlines on social media: Effects of truth-bias and endorsement cues. *Communication Research,**49*(2), 171–195. 10.1177/0093650220921321

[CR38] MacFarlane, D., Tay, L. Q., Hurlstone, M. J., & Ecker, U. K. H. (2021). Refuting spurious COVID-19 treatment claims reduces demand and misinformation sharing. *Journal of Applied Research in Memory and Cognition,**10*(2), 248–258. 10.1016/j.jarmac.2020.12.005 PMC7771267 33391983

[CR39] Maertens, R., Götz, F. M., Golino, H. F., Roozenbeek, J., Schneider, C. R., Kyrychenko, Y., Kerr, J. R., Stieger, S., McClanahan, W. P., Drabot, K., He, J., & van der Linden, S. (2024). The Misinformation Susceptibility Test (MIST): A psychometrically validated measure of news veracity discernment. *Behavior Research Methods,**56*(3), 1863–1899. 10.3758/s13428-023-02124-2 PMC10991074 37382812

[CR40] McPhedran, R., Ratajczak, M., Mawby, M., King, E., Yang, Y., & Gold, N. (2023). Psychological inoculation protects against the social media infodemic. *Scientific Reports,**13*(1), 5780–5780. 10.1038/s41598-023-32962-1 PMC10082776 37031339

[CR41] Modirrousta-Galian, A., Higham, P. A., & Seabrooke, T. (2023). Effects of inductive learning and gamification on news veracity discernment. *Journal of Experimental Psychology: Applied,**29*(3), 599–619. 10.1037/xap0000458 36862469

[CR42] Modirrousta-Galian, A., Higham, P. A., & Seabrooke, T. (2025). Wordless wisdom: The dominant role of tacit knowledge in true and fake news discrimination. *Journal of Applied Research in Memory and Cognition,**14*(2), 231–240. 10.1037/mac0000151

[CR43] Modirrousta-Galian, A., Seabrooke, T., Hanoch, Y., Kelley, N. J., & Higham, P. A. (2026). An inductive learning intervention to improve news veracity discernment. *Journal of Experimental Psychology: Applied*. 10.1037/xap0000580 42113132

[CR44] Molina, M. D., Sundar, S. S., Le, T., & Lee, D. (2021). “Fake news” is not simply false information: A concept explication and taxonomy of online content. *American Behavioral Scientist,**65*(2), 180–212. 10.1177/0002764219878224

[CR45] Murphy, G., Loftus, E. F., Grady, R. H., Levine, L. J., & Greene, C. M. (2019). False memories for fake news during Ireland’s abortion referendum. *Psychological Science,**30*(10), 1449–1459. 10.1177/0956797619864887 31432746

[CR46] Murphy, G., de Saint Laurent, C., Reynolds, M., Aftab, O., Hegarty, K. Sun, Y. & Greene, C. M. (2023). What do we study when we study misinformation?: A scoping review of experimental research (2016–2022). *Harvard Kennedy School (HKS) Misinformation Review, 4*(6)

[CR47] NASA Science (2024, October 21). *Scientific consensus*. https://science.nasa.gov/climate-change/scientific-consensus/

[CR48] Orchinik, R., Martel, C., Rand, D., & Bhui, R. (2025). *Adaptive intuitions shape susceptibility to misinformation*. PsyArXiv. 10.31234/osf.io/q7r58_v2

[CR49] Pennycook, G., & Rand, D. G. (2020). Who falls for fake news? The roles of bullshit receptivity, overclaiming, familiarity, and analytic thinking. *Journal of Personality,**88*(2), 185–200. 10.1111/jopy.12476 30929263

[CR50] Pehlivanoglu, D., Lin, T., Deceus, F., Heemskerk, A., Ebner, N. C., & Cahill, B. S. (2021). The role of analytical reasoning and source credibility on the evaluation of real and fake full-length news articles. *Cognitive Research: Principles and Implications,**6*(1), 24. 10.1186/s41235-021-00292-3 PMC8012428 33788040

[CR51] Pennycook, G., Binnendyk, J., Newton, C., & Rand, D. G. (2021). A practical guide to doing behavioral research on fake news and misinformation. *Collabra: Psychology*. 10.1525/collabra.25293

[CR52] Pennycook, G., & Rand, D. G. (2019). Lazy, not biased: Susceptibility to partisan fake news is better explained by lack of reasoning than by motivated reasoning. *Cognition,**188*, 39–50. 10.1016/j.cognition.2018.06.011 29935897

[CR53] Pennycook, G., & Rand, D. G. (2022). Accuracy prompts are a replicable and generalizable approach for reducing the spread of misinformation. *Nature Communications,**13*, 2333. 10.1038/s41467-022-30073-5 PMC9051116 35484277

[CR54] Pennycook, G., & Rand, D. G. (2021). The psychology of fake news. *Trends in Cognitive Sciences,**25*(5), 388–402. 10.1016/j.tics.2021.02.007 33736957

[CR55] Perach, R., Joyner, L., Husbands, D., & Buchanan, T. (2023). Why do people share political information and misinformation online? Developing a bottom-up descriptive framework. *Social Media & Society*. 10.1177/20563051231192032

[CR56] Pereira, A., Harris, E., & Van Bavel, J. J. (2023). Identity concerns drive belief: The impact of partisan identity on the belief and dissemination of true and false news. *Group Processes & Intergroup Relations,**26*(1), 24–47. 10.1177/13684302211030004

[CR57] Pexels. (2025) *Terms.* https://www.pexels.com/terms-of-service/

[CR58] Pfänder, J., & Altay, S. (2025). Spotting false news and doubting true news: A systematic review and meta-analysis of news judgements. *Nature Human Behaviour,**9*(4), 688–699. 10.1038/s41562-024-02086-1 PMC12018262 39984640

[CR59] Roozenbeek, J., Lasser, J., Marks, M., Qin, T., Garcia, D., Goldberg, B., Debnath, R., van der Linden, S., & Lewandowsky, S. (2025). *Misinformation interventions and online sharing behavior: lessons learned from two preregistered field studies*. PsyArXiv. 10.31234/osf.io/upvyx_v2 PMC12646748 41307058

[CR60] Roozenbeek, J., Traberg, C. S., & van der Linden, S. (2022a). Technique-based inoculation against real-world misinformation. *Royal Society Open Science,**9*(5), 211719. 10.1098/rsos.211719 PMC9114974 35600423

[CR61] Roozenbeek, J., van der Linden, S., Goldberg, B., Rathje, S., & Lewandowsky, S. (2022). Psychological inoculation improves resilience against misinformation on social media. *Science Advances,**8*(34), Article eabo6254. 10.1126/sciadv.abo6254 PMC9401631 36001675

[CR62] Schincariol, A., Otgaar, H., Greene, C. M., Murphy, G., Riesthuis, P., Mangiulli, I., & Battista, F. (2024). Fake memories: A meta-analysis on the effect of fake news on the creation of false memories and false beliefs. *Memory, Mind & Media,**3*, e17. 10.1017/mem.2024.14

[CR63] Shephard, M. P., Robertson, D. J., Huhe, N., & Anderson, A. (2023). Everyday non-partisan fake news: Sharing behavior, platform specificity, and detection. *Frontiers in Psychology,**14*, 1118407. 10.3389/fpsyg.2023.1118407 PMC10225687 37255519

[CR64] Shen, C., Kasra, M., Pan, W., Bassett, G. A., Malloch, Y., & O’Brien, J. F. (2019). Fake images: The effects of source, intermediary, and digital media literacy on contextual assessment of image credibility online. *New Media & Society,**21*(2), 438–463. 10.1177/1461444818799526

[CR65] Simons, D. J., Shoda, Y., & Lindsay, D. S. (2017). Constraints on generality (COG): A proposed addition to all empirical papers. *Perspectives on Psychological Science,**12*(6), 1123–1128. 10.1177/1745691617708630 28853993

[CR66] Smith, R., Chen, K., Winner, D., Friedhoff, S., & Wardle, C. (2023). A systematic review of COVID-19 misinformation interventions: Lessons learned. *Health Affairs,**42*(12), 1738–1746. 10.1377/hlthaff.2023.00717 37967291

[CR67] Spring, M. (2024, August 8). *The real story of the news website accused of fuelling riots*. BBC News. https://www.bbc.co.uk/news/articles/c5y38gjp4ygo

[CR68] Springer Nature (2025). *Third-party permissions*. https://www.springernature.com/gp/policies/editorial-policies/third-party-permissions

[CR69] Sun, Y., & Xie, J. (2024). Who shares misinformation on social media? A meta-analysis of individual traits related to misinformation sharing. *Computers in Human Behavior,**158*, 108271. 10.1016/j.chb.2024.108271

[CR70] Tandoc, E. C., Lim, Z. W., & Ling, R. (2018). Defining “fake news”: A typology of scholarly definitions. *Digital Journalism,**6*(2), 137–153. 10.1080/21670811.2017.1360143

[CR71] The University of Cambridge, BBC Media Action, & Jigsaw (2022). *A practical guide to prebunking misinformation*. https://prebunking.withgoogle.com/docs/A_Practical_Guide_to_Prebunking_Misinformation.pdf

[CR72] Traberg, C. S., Roozenbeek, J., & van der Linden, S. (2022). Psychological inoculation against misinformation: Current evidence and future directions. *The Annals of the American Academy of Political and Social Science,**700*(1), 136–151. 10.1177/00027162221087936

[CR73] Tricco, A. C., Lillie, E., Zarin, W., O’Brien, K. K., Colquhoun, H., Levac, D., Moher, D., Peters, M. D. J., Horsley, T., Weeks, L., Hempel, S., Akl, E. A., Chang, C., McGowan, J., Stewart, L., Hartling, L., Aldcroft, A., Wilson, M. G., Garritty, C., & Straus, S. E. (2018). PRISMA extension for scoping reviews (PRISMA-ScR): Checklist and explanation. *Annals of Internal Medicine,**169*(7), 467–473. 10.7326/M18-0850 30178033

[CR74] Uscinski, J. (2023). What are we doing when we research misinformation? *Political Epistemology*, *2*, 2-14. https://www.academia.edu/97403059/Political_Epistemology_No_2

[CR75] Uscinski, J., Littrell, S., & Klofstad, C. (2024). The importance of epistemology for the study of misinformation. *Current Opinion in Psychology,**57*, Article 101789. 10.1016/j.copsyc.2024.101789 38301573

[CR76] van der Linden, S., & Roozenbeek, J. (2021, January 19). *COVID-19 misinformation: scientists create a ‘psychological vaccine’ to protect against fake news*. The Conversation. https://theconversation.com/covid-19-misinformation-scientists-create-a-psychological-vaccine-to-protect-against-fake-news-153024

[CR77] Walton, A. G. (2017, January 23). *‘Psychological vaccine’ may protect against fake news, alternative facts*. Forbes. https://www.forbes.com/sites/alicegwalton/2017/01/23/psychological-vaccine-may-protect-against-fake-news-alternative-facts/

[CR78] Wang, S. Y. N., Phillips, S. C., Carley, K. M., Lin, H., & Pennycook, G. (2025). Limited effectiveness of psychological inoculation against misinformation in a social media feed. *PNAS Nexus*. 10.1093/pnasnexus/pgaf172 PMC12134459 40470142

[CR79] Wenjuan, L., Zhaotong, Y., Yuhua, D., & Midi, Z. (2023). The influence of involvement and emotional valence on accuracy judgments and sharing intention of fake news. *Journal of Cognitive Psychology,**35*(8), 839–855. 10.1080/20445911.2023.2241699

[CR80] Yan, W., & Pan, Z. (2023). Believing and sharing false news on social media: The role of news presentation, epistemic motives, and deliberative thinking. *Media Psychology,**26*(6), 743–766. 10.1080/15213269.2023.2208363

[CR81] Yoo, J. H. (2025). On the controversies surrounding the lab-leak theory of COVID-19. *Journal of Korean Medical Science*. 10.3346/jkms.2025.40.e153 PMC12040609 40296830

